# Super-Resolution and Feature Extraction for Ocean Bathymetric Maps Using Sparse Coding

**DOI:** 10.3390/s22093198

**Published:** 2022-04-21

**Authors:** Taku Yutani, Oak Yono, Tatsu Kuwatani, Daisuke Matsuoka, Junji Kaneko, Mitsuko Hidaka, Takafumi Kasaya, Yukari Kido, Yoichi Ishikawa, Toshiaki Ueki, Eiichi Kikawa

**Affiliations:** 1 Research Institute for Marine Geodynamics (IMG), Japan Agency for Marine-Earth Science and Technology (JAMSTEC), 2-15 Natsushima-cho, Yokosuka 237-0061, Japan; taku_yutani@jamstec.go.jp; 2 Research Institute for Value-Added-Information Generation (VAiG), Japan Agency for Marine-Earth Science and Technology (JAMSTEC), 3173-25 Showa-machi, Isogo-ku, Yokohama 236-0001, Japan; daisuke@jamstec.go.jp (D.M.); mitsukou@jamstec.go.jp (M.H.); ishikaway@jamstec.go.jp (Y.I.); kikawa@jamstec.go.jp (E.K.); 3 Ocean High Technology Institute, Inc., 2-29-12 Honcho, Nakano-ku, Tokyo 164-0012, Japan; yono@ohti.co.jp (O.Y.); ueki@ohti.co.jp (T.U.); 4 Research Institute for Marine Resources Utilization, Japan Agency for Marine-Earth Science and Technology (JAMSTEC), 2-15 Natsushima-cho, Yokosuka 237-0061, Japan; kanekoj@jamstec.go.jp (J.K.); tkasa@jamstec.go.jp (T.K.); 5 Institute for Marine-Earth Exploration and Engineering (MarE3), Japan Agency for Marine-Earth Science and Technology (JAMSTEC), 2-15 Natsushima-cho, Yokosuka 237-0061, Japan; ykido@jamstec.go.jp; 6 IDEA Consultants, Inc., 2-2-2 Hayabuchi, Tsuzuki-ku, Yokohama 224-0025, Japan

**Keywords:** bathymetric map, super-resolution, dictionary learning, sparse modelling, image processing

## Abstract

The comprehensive production of detailed bathymetric maps is important for disaster prevention, resource exploration, safe navigation, marine salvage, and monitoring of marine organisms. However, owing to observation difficulties, the amount of data on the world’s seabed topography is scarce. Therefore, it is essential to develop methods that effectively use the limited data. In this study, based on dictionary learning and sparse coding, we modified the super-resolution technique and applied it to seafloor topographical maps. Improving on the conventional method, before dictionary learning, we performed pre-processing to separate the teacher image into a low-frequency component that has a general structure and a high-frequency component that captures the detailed topographical features. We learn the topographical features by training the dictionary. As a result, the root-mean-square error (RMSE) was reduced by 30% compared with bicubic interpolation and accuracy was improved, especially in the rugged part of the terrain. The proposed method, which learns a dictionary to capture topographical features and reconstructs them using a dictionary, produces super-resolution with high interpretability.

## 1. Introduction

Ocean bathymetric maps provide basic information in various scientific and engineering fields, including geomorphology, physical oceanography, disaster prevention, and resource exploration. Despite its importance, more than three-quarters of the total ocean floor on Earth remains unmapped using detailed measurement methods such as acoustic surveys with a 15 arc-second (~250 m) interval grid, which is the reference resolution of the GEBCO_2021 Grid [1]. Because it is recognised as a global issue, several international and domestic projects, such as the “Nippon Foundation-GEBCO SEABED2030 Project (SEABED2030)” [2] and “DeSET Project” [3], is currently underway. Creating topographical maps with as high resolution as possible from available datasets and finding characteristic topographical patterns from them solves various problems. The technology to generate high-resolution (HR) from low-resolution (LR) seafloor topographical maps can be a supplementary means when comprehensive and detailed acoustic surveys using ships are difficult.

Super-resolution is a general term for image processing, such as upscaling and/or improving the image details. The simplest and straightforward method for upscaling an image uses geometrical interpolation, such as bilinear and bicubic methods. Conversely, this approach only uses information about the continuity of pixel value, which is a mainstream approach in the image-processing field, and uses useful information on the details of images by presumably learning the correspondence between LR and HR image pairs (example, [4,5,6,7,8]). The approach includes sparse-coding methods (example, [4,6,9,10,11,12]), that use the property of images in which small patches from images can be represented by the sum of a small number of image bases and deep-learning methods that enable complex feature extraction contained in data by combining multiple layers and numerous feature-extraction filters (example, [13,14,15]).

Recently, deep-leaning-type methods have been applied to the super-resolution of bathymetric maps (example, [16,17,18]), and they have attracted considerable attention owing to their high prediction accuracy, which exceeds that of conventional interpolation methods (example, [19]). However, they have several drawbacks in terms of applications to scientific problems [20]. (1) They require numerous datasets which are appropriate for a specific target problem, and (2) they are highly black-boxed and have low interpretability which is not suitable for obtaining scientific knowledge. Therefore, it is expected to develop a high-resolution method with interpretability that allows the results to be simply understood and leads to the derivation of scientific knowledge.

In this study, we focus on the potential of sparse coding super-resolution (ScSR) based on dictionary learning. This method is highly interpretable because it is a simple linear method for super-resolution which extracts a small number of important features. Therefore, the application of ScSR to seabed topographical maps is expected to simultaneously provide highly interpretable topographical-feature extraction and super-resolution. The objective of this study is to establish a super-resolution method for ocean bathymetric images using sparse modelling and verify its usefulness for increasing the resolution of rough bathymetry and extracting features of seafloor topography.

We describe the method, which is mainly based on Yang’s and Elad’s methods [6,9]; however, it is generally extended for use in natural science data. Subsequently, the method is applied to a multibeam echo sounder (MBES) from the Mid-Okinawa Trough. The results are compared with the bicubic method as a standard method and improvements in the accuracy and extraction of topographical features are discussed. This is a preliminary report on the incubation stag; the future direction of research is discussed.

## 2. Method

**Figure 1:**
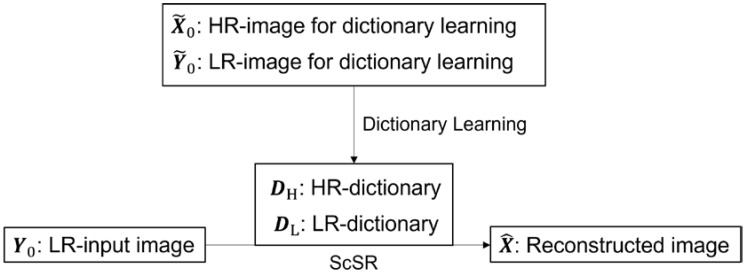
Flowchart of the outline of ScSR. HR and LR indicate high-resolution and low-resolution, respectively.

**Figure 2:**
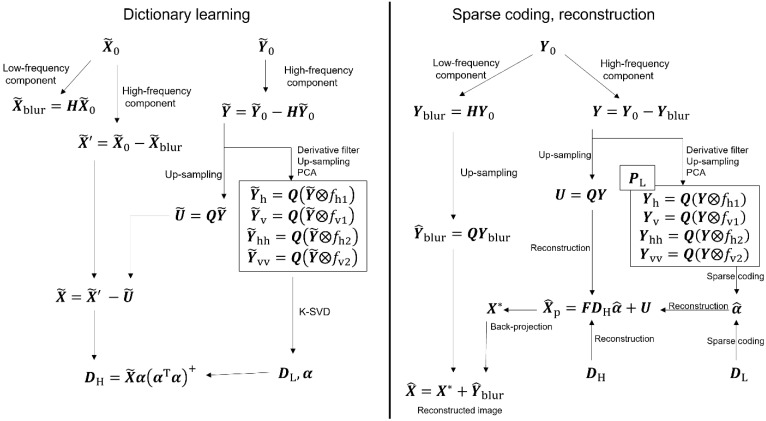
Detailed flowchart of ScSR. It consists of two processes: dictionary learning and reconstruction by sparse coding.

In this section, the core algorithm for the super-resolution of seabed topography is explained. The proposed algorithm, which is mainly based on [9], consists of three parts—dictionary learning, sparse coding, and reconstruction (Figure 1 and Figure 2).

### 2.1. Dictionary Learning

This subsection describes the data pre-processing procedures and the algorithms for dictionary learning. Important features in bathymetry often involve abrupt changes in depth. In order to extract topographical features from bathymetric maps, high-frequency components were extracted from original images by separating low-frequency components. The low-frequency component ${\tilde{X}}_{\mathrm{blur}}$ was obtained by applying a Gaussian filter to ${\tilde{X}}_{0}$, which is the original HR image for dictionary learning. The subtraction of ${\tilde{X}}_{\mathrm{blur}}$ from ${\tilde{X}}_{0}$ yields the high-frequency component ${\tilde{X}}^{\prime }$ using the following equation.

(1) $${\tilde{X}}^{\prime }={\tilde{X}}_{0}-{\tilde{X}}_{\mathrm{blur}}.$$

This is the target of sparse-coding super-resolution (ScSR) estimation. Furthermore, $\tilde{Y}$ is the high-frequency component of the LR grid data ${\tilde{Y}}_{0}$, which is the original LR image used for dictionary learning.

(2) $$\tilde{Y}={\tilde{Y}}_{0}-H{\tilde{Y}}_{0},$$

where H is the Gaussian filter used as a blurring operator. The Gaussian filter is an averaging filter weighted according to the spatial Gaussian distribution.

Edge components of $\tilde{Y}$ is extracted and divided into N patches. See Appendix A for details on edge component extraction of $\tilde{Y}$ and its dimensionality reduction. The length of patches of each edge component is compressed to $n_{\mathrm{low}}$.

The LR dictionary $D_{L}$ is learned by applying the K-SVD algorithm [21] on the edge component patches of $\tilde{Y}$. For the obtained patch set with $n_{\mathrm{low}}$ patch length, the K-SVD algorithm is used to solve the following optimisation problem to learn an LR dictionary $D_{L}\in {\mathbb{R}}^{n_{\mathrm{low}}\times N_{\mathrm{atom}}}$, where $N_{\mathrm{atom}}$ is the number of atoms in the dictionary.

(3) $$D_{L}=\arg\underset{D,\alpha }{\min}\|P-D\alpha {\|}_{2}^{2}\;\mathrm{subject}\;\mathrm{to}\;\forall i,\;\|\alpha_{i}{\|}_{0}\leq k_{0},$$

where $P\in {\mathbb{R}}^{n_{\mathrm{low}}\times N}$ is a matrix with each patch as a column element, $\alpha \in {\mathbb{R}}^{N_{\mathrm{atom}}\times N}$ is a matrix with sparse code $\alpha_{i}$ as a column element corresponding to the *i*-th patch, and $k_{0}$ is the maximum number of non-zero elements. The learning process began by fixing the initial dictionary D and finding α using orthogonal matching pursuit (OMP). Then, α is fixed and dictionary D is updated using the K-SVD algorithm. The initial dictionary D was randomly sampled from the standard normal distribution, and the column was normalised.

To learn the HR dictionary $D_{H}$ from $X^{\prime }$, we focus on the edge component of $X^{\prime }$ and generate the difference data $\tilde{X}$ between ${\tilde{X}}^{\prime }$ and the low-frequency component $\tilde{U}\left(=Q\tilde{Y}\right)$,

(4) $$\tilde{X}={\tilde{X}}^{\prime }-\tilde{U},$$

where Q is an up-sampling operator. In this study, we adopted bicubic interpolation as the up-sampling method. Bicubic interpolation smoothly interpolates luminance values by fitting them with a cubic function using four pixels around the target coordinates.

The HR dictionary $D_{H}$ is created using HR learning data $\tilde{X}$ and the sparse representation matrix ***α*** obtained by learning the LR dictionary $D_{L}$,

(5) $$D_{H}=\tilde{X}\alpha {\left(\alpha^{T}\alpha \right)}^{+},$$

where ${\left(\alpha^{T}\alpha \right)}^{+}$ is the Moore-Penrose pseudo-inverse [22,23] of $\alpha^{T}\alpha$.

### 2.2. Sparse-Coding and Reconstruction

The obtained LR image $Y_{0}$ is separated into a low-frequency component $Y_{\mathrm{blur}}$, and a high-frequency component Y.

(6) $$Y_{\mathrm{blur}}=HY_{0},$$

(7) $$Y=Y_{0}-Y_{\mathrm{blur}}.$$

We extracted the edge component of the high-frequency component Y by applying a differential filter in the same manner as in the dictionary learning process, and patch length of edge components of Y is compressed to $n_{L}$ by PCA (see Appendix A).

Using the learned $D_{L}$ for matrix $P_{L}\in {\mathbb{R}}^{n_{L}\times N}$ with the obtained patches as the edge components of Y, we obtained matrix $\hat{\alpha }\in {\mathbb{R}}^{N_{\mathrm{atom}}\times N}$ with the corresponding sparse code ${\hat{\alpha }}_{i}$ as column elements. To solve this, we compute the following optimisation problem in the OMP [24]:

(8) $$\hat{\alpha }=\arg\underset{\;\alpha }{\min}\|P_{L}-D_{L}\alpha {\|}_{2}^{2}\;\;\;\mathrm{subject}\;\mathrm{to}\;\forall i,\;\|\alpha_{i}{\|}_{0}\leq k_{0}.$$

To reconstruct an HR image, we used $\hat{\alpha }$ obtained in the sparse coding process and the HR dictionary $D_{H}$. We obtained matrix ${\hat{P}}_{H}$ with a group of HR patches as column elements from the product of the obtained sparse representation matrix $\hat{\alpha }$ and LR dictionary $D_{L}$,

(9) $${\hat{P}}_{H}=D_{H}\hat{\alpha }.$$

The HR patch data ${\hat{X}}_{p}$ is reconstructed by adding U(=QY) to a matrix which is generated by stitching the set of patches ${\hat{P}}_{H}$ (Figure 2).

(10) $${\hat{X}}_{p}=F{\hat{P}}_{H}+U,$$

where F is an operator that superposes the adjacent patches and takes the average value of the overlap region. Subsequently, the patch data ${\hat{X}}_{p}$ is refined to $X^{*}$ by back projection, as proposed by [6]. Back-projection algorithm constrains the difference between the input LR image Y and a reconstructed LR image $D_{L}\hat{\alpha }$, which is not taken into account during the process of reconstructing an HR image. The refined image $X^{*}$ is obtained by computing

(11) $$X^{*}=\arg\underset{X}{\min}\|SHX-Y{\|}_{2}^{2},$$

where S is a down-sampling operator.

Finally, the $Y_{\mathrm{blur}}$, which was initially removed as a low-frequency component, is up-sampled by cubic interpolation and combined to form the final reconstructed grid data $\hat{X}$,

(12) $$\hat{X}=X^{*}+{\hat{Y}}_{\mathrm{blur}}.$$

The algorithm structure of sparse coding and reconstruction is written below (Algorithm 1). We used Python for data pre-processing, dictionary learning, sparse coding, reconstruction, and visualisation of figures, and GMT for visualisation of the original bathymetric maps.
**Algorithm 1**. Reconstruction algorithm for sparse coding super-resolution (ScSR).0: Learn HR and LR dictionaries, $D_{H}$ and $D_{L}$ 1: **Input**: dictionaries, $D_{H}$ and $D_{L}$, edge components of an LR image Y 2: Split an LR image $Y_{0}$ into high- and low-frequency components, Y and $Y_{\mathrm{blur}}$. 3: Extract LR patches $P_{L}$ from the edge components of Y. 4:      $\hat{\alpha }\leftarrow SparseCoding\left(D_{L},\;P_{L}\right)$ 5:      Generate the HR patch: ${\hat{P}}_{H}\leftarrow D_{H}\hat{\alpha }$. 6:      Up-sample the high-frequency component of the LR image, U←QY 7:   Superpose the adjacent patches and add U: ${\hat{X}}_{p}\leftarrow F{\hat{P}}_{H}+U.$ 8:   Find $X^{*}$ which satisfies the constraint: $X^{*}=\arg\underset{X}{\min}\|SHX-Y{\|}_{2}^{2}$. 9:  Up-sample the low-frequency component of the LR image: ${\hat{Y}}_{\mathrm{blur}}\leftarrow QY_{\mathrm{blur}}$. 10: Take a summation of reconstructed component $X^{*}$ and up-sampled component ${\hat{Y}}_{\mathrm{blur}}$: $\hat{X}\leftarrow X^{*}+{\hat{Y}}_{\mathrm{blur}}$. 11: **Output**: SR image $\hat{X}$.

## 3. Data and Implementation

**Figure 3:**
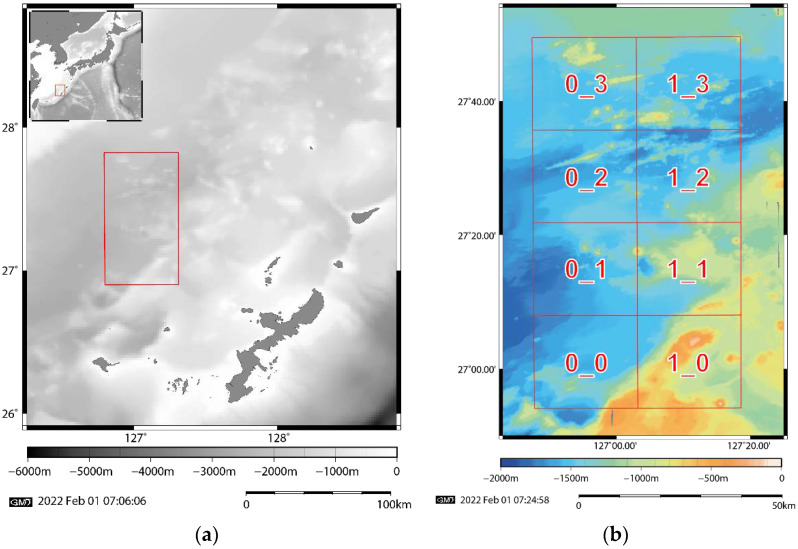
Map of the area used in this study. (**a**) The Japanese Islands (top left) and the sea around Okinawa; (**b**) Topographical map in the red box in (**a**). The area is equally divided into eight squares. The ridge in the area 0_2 is the Iheya–Minor Ridge.

To verify the effectiveness of super-resolution by dictionary learning, we used bathymetry data from the Mid-Okinawa Trough (Figure 3), where the Iheya–Minor Ridge, small sea knolls, and faults associated with the Okinawa Trough have been identified [25,26,27]. Pairs of HR and LR grid data that require increased resolution of the area are used as training datasets. The target resolution data was a mesh grid of 50-m intervals drawn by calculation of the grid data from point clouds of water depths in the Mid-Okinawa Trough obtained by multiple types of MBES [28].

The bathymetry data were normalised to the required extent before processing. The depth range of the input data was altitudes of −3000–0 m in this study. This was normalised to a range of 0–1 for training purposes. The original data ${\tilde{X}}_{0}$ has a 50-m bathymetric grid, which is a target resolution in this study. During the dictionary learning process, LR images were required to obtain an LR dictionary. The following process is applied to ${\tilde{X}}_{0}$ to obtain LR grid data ${\tilde{Y}}_{0}$ for super-resolution,

(13) $${\tilde{Y}}_{0}=SH{\tilde{X}}_{0},$$

where S is a down-sampling operator, that is, the data to be super-resolved in this study is an LR image obtained by down-sampling 50-m grid data to 100-m grid data. The obtained pairs of HR and LR images were divided into eight 25.6 km squares (Figure 3), and dictionary learning was performed in each area. Using these eight pairs of dictionaries, we performed super-resolution with ScSR in the other seven areas.

As mentioned above, the selected area of the Mid-Okinawa Trough was divided into eight sections (Figure 3); eight dictionaries were created by dictionary learning using the K-SVD method in each area and the accuracy of each dictionary was validated in the remaining seven areas. In this study, dictionary learning was conducted with the following hyper-parameters: the number of bases was 256, the maximum number of non-zero elements was 2, the patch size was 16 × 16, and the image was reconstructed with stride 2. We set the values of these parameters based on the preliminary experimental results. Patch size (16) corresponds to an actual length of 800 m. The root-mean-square error (RMSE) relative to an original image was used to evaluate accuracy. We reconstructed the remaining seven regions using eight dictionaries generated by dictionary learning. To verify the effectiveness of ScSR, we applied bicubic interpolation to LR grid data for comparison.

## 4. Results

**Figure 4:**
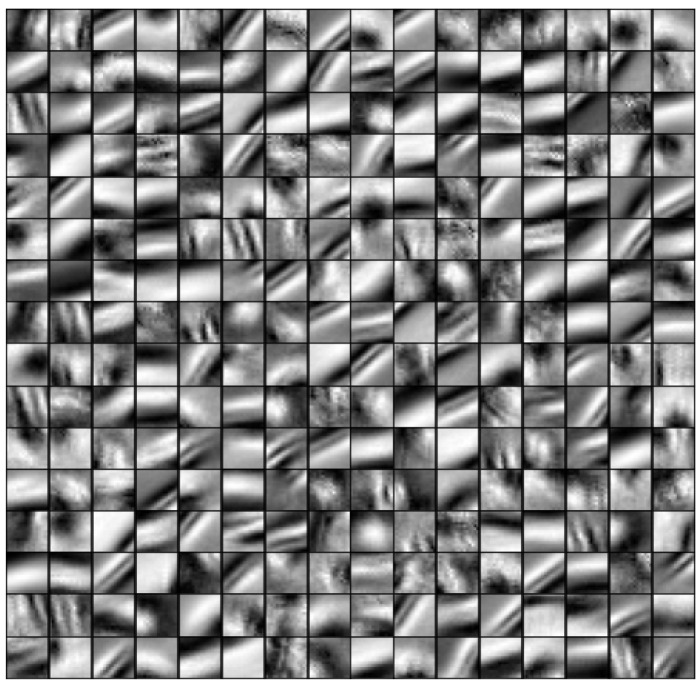
High-resolution image patch of high-frequency component trained with the area 0_0.

**Figure 5:**
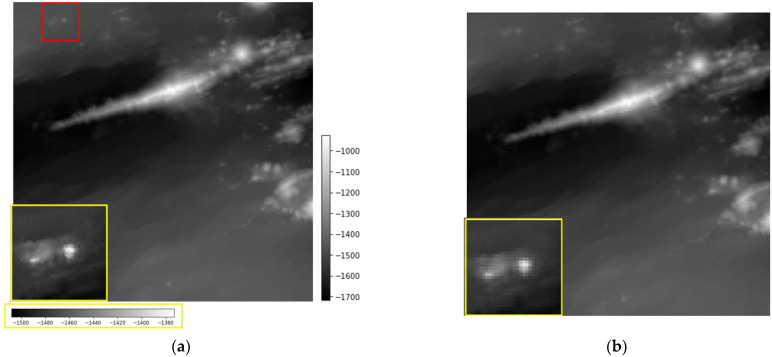

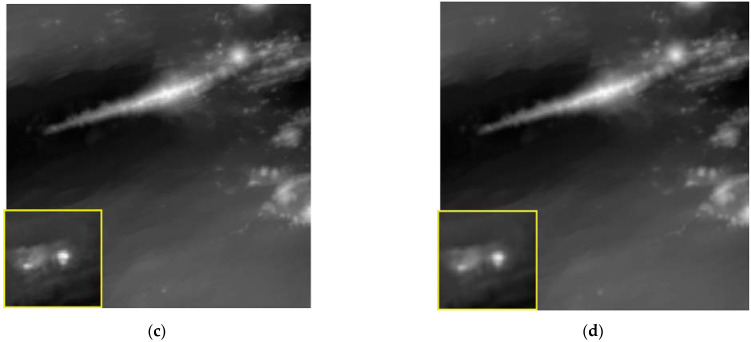
Seabed topography in area 0_2. (**a**) The original image; (**b**) Low-resolution input image; (**c**) Sparse coding super-resolution (ScSR: the proposed method); (**d**) Bicubic interpolation.

The results of the dictionary which were learned in area 0_0 (Figure 4) and the image of area 0_2, which was reconstructed using dictionary 0_0, are presented. We obtained a sparse representation matrix α which approximates the LR image of area 0_2 using dictionary 0_0. α and HR dictionary $D_{H}$ were used to reconstruct the HR image in 0_2 (Figure 5). The bases within the dictionary of 0_0 show that the learning process extracts variable geomorphic characteristics, such as ridges or valley-like and mountain-like shapes (Figure 4). Details of the topographical features indicated by the bases in the dictionary are discussed in the “Discussion” section.

**Figure 6:**
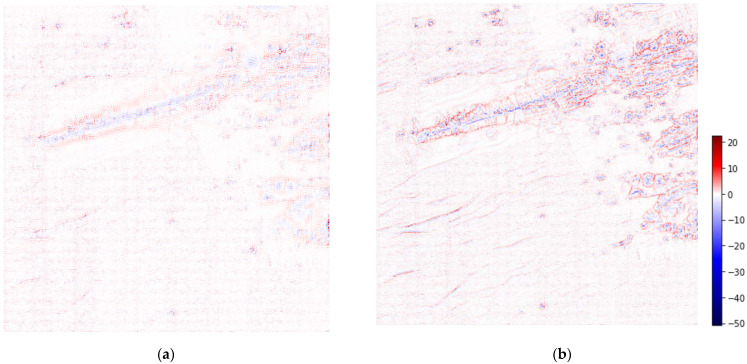
Residual images between the super-resolution images and original image of the area 0_2. (**a**) ScSR (RMSE: 1.157 m); (**b**) Bicubic interpolation (RMSE: 1.713 m). The same colour scale is used in both images.

The 0_2 image reconstructed with ScSR shows the topographical structure more clearly than the bicubic interpolation. Focusing on the eastern part of the Iheya–Minor Ridge, the ScSR image (Figure 5c) shows the topographical undulations more clearly compared to the LR (Figure 5b) and bicubic interpolation images (Figure 5d). In addition, in the western part of the Iheya–Minor Ridge, bicubic interpolation introduces undesired smoothing, that is not present in our proposed method. Figure 6 shows the residual images between the original HR image and bicubic-interpolated and reconstructed ScSR images. The bicubic interpolation image has large errors in large undulating areas, such as the Iheya–Minor Ridge, small knolls, and faults, whereas the ScSR has small errors.

The RMSE of the ScSR image was 1.156 m, while that of the bicubic image was 1.713 m. Table 1 presents the RMSEs for the relevant regions reconstructed using the dictionaries learned in the other seven areas. The RMSEs of bicubic interpolation are shown in the table. The RMSE of the ScSR is approximately 30% lower than that of bicubic interpolation in all the regions, indicating that ScSR improves the accuracy.

## 5. Discussion

Because each basis was a feature extracted from the seafloor topography of the training dataset, the basis used in the reconstruction was chosen to represent the features of the topography of the corresponding area. The basis used in this study is an 800 m square, which is suitable for extracting geomorphological features, such as small sea knolls. In this section, we verify the characteristics of the bases which are learned in 0_0 and choose to reconstruct area 0_2 and verify if the topographical features in this area are extracted properly.

**Figure 7:**
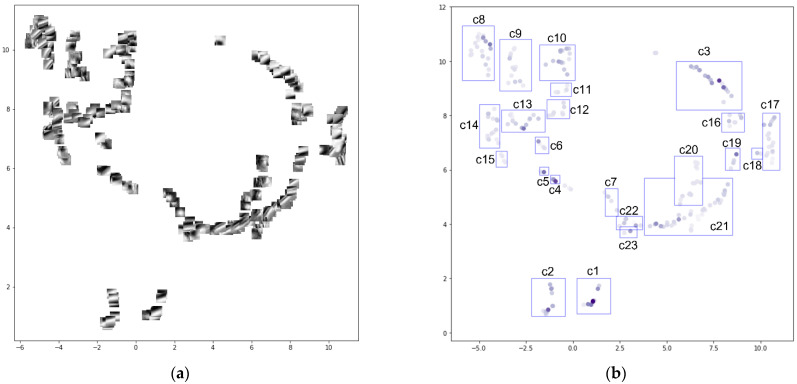
(**a**) Visualisation of 256 bases from the “0_0” dictionary as embedded by UMAP. (**b**) Distribution of clusters of bases on the embedded space by UMAP. The colour shading of the symbols on (**b**) corresponds to the sum of the absolute values of the coefficients of each basis in the reconstruction. “c1” represents “cluster 1”, and the same applies to “c2” and beyond.

Focusing on the topographical features of each basis, some bases in the 0_0 dictionary show similar patterns to each other. To simplify the interpretation, we performed uniform manifold approximation and projection (UMAP) [29] to project similar bases close to each other and classified them into 23 groups based on the results (Figure 7). Figure 7 shows that the dictionary contains bases with ridges and valleys extending in the ENE-SWS direction (cluster 1 and 21), bases with small basin-like or mountain-like shapes in the central part (cluster 7 and 11), bases with the NE-SW ridge features (cluster 8), and bases with a ridge extending in the NS (cluster 14). Area 0_2 includes the Iheya–Minor Ridge of the Mid-Okinawa Trough. Several faults running in the ENE-SWS direction were identified to the south of the ridge. Small sea knolls were also observed in the centre of the southern part of the area and north of the ridge.

**Figure 8:**
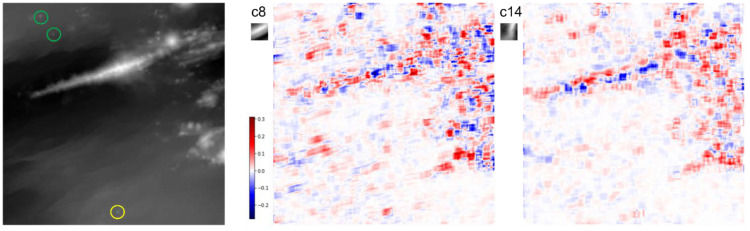
Distribution of clusters 8 and 14 based on the clustering (Figure 7) in the reconstructed area. The small figure to the top left of the coloured maps shows the representative basis within the cluster, that is, the one with the sum of the absolute values of the coefficients throughout the reconstruction is the largest in each cluster. The left figure shows the original image of the reconstructed area. The geological edifices in the green and yellow circles are the small sea knolls. See text for details.

**Figure 9:**
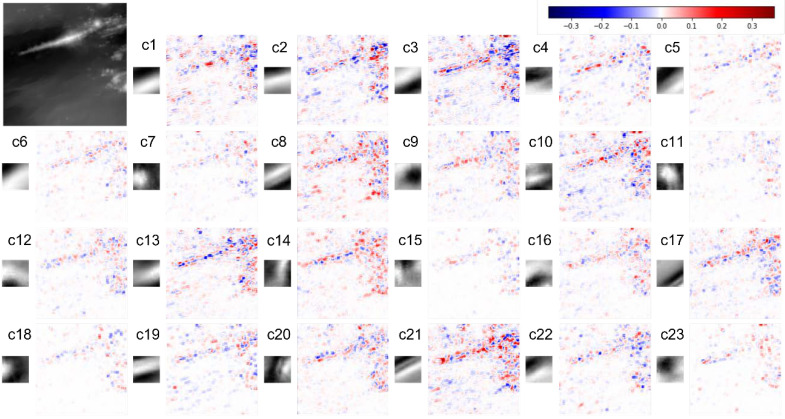
Distribution of the 23 clusters in the reconstructed area. The small figure to the left of each map shows the representative basis of the cluster. The scale of the colour bar is identical to that shown in Figure 8.

We examined the extraction of the geomorphic features represented by the bases selected during the reconstruction of the Iheya–Minor Ridge, faults, and small sea knolls. Figure 8 and Figure 9 show the contribution of each group to the reconstruction of area 0_2. Figure 8 shows that the absolute values of the coefficients of the bases are larger around the faults and Iheya–Minor Ridge. Focusing on the bases used to reconstruct the small sea knolls, different groups of bases were extracted for each small knoll. Specifically, cluster 14, which is represented by the basis of a ridge extending in the NS direction (Figure 7), is used for the sparse representation of two small knolls north of the Iheya–Minor Ridge (enclosed by green circles in Figure 8); however, it is not used for the representation of the knoll in the southern part of the region (within a yellow circle), which is located more than 10 km away from the ridge (Figure 8). Conversely, cluster 8, which captures the NE-SW ridge features, is used for sparse representation of the southern knoll and its surrounding faults but is not used for reconstruction of the northern knoll (Figure 8). Reconstructing an HR image using sparse representation probably enables the capture of topographical features that are not apparent to the naked eye.

The accuracy of super-resolution detail was also examined using the residual map of area 0_2 (Figure 6). As mentioned above, steep gradients, which are often features of geoscientific importance, are not well represented in the residual map of simple interpolation methods (Figure 6b). Conversely, ScSR shows a significant improvement in errors at the Iheya–Minor Ridge, small knolls, and faulted areas (Figure 6a). The proposed method selects the basis while constraining the reconstruction to capture topographical features at the patch-size scale.

Variations in accuracy for each region are also discussed in terms of topographical features. Focusing on the RMSE of the bicubic interpolation, the value remarkably varies from one area to another (Table 1). Accuracy in bicubic interpolation is better in relatively gentle and uniform sea areas, e.g., 0_0 and 0_1. On the other hand, the bicubic interpolation accuracy is poor for other sea areas where local topographical changes are notably recognised, such as small ridges and sea hills (Figure 3). In terms of the accuracy of ScSR, we will discuss in which areas ScSR is highly effective compared to bicubic interpolation. A comparison of the RMSEs of ScSR and bicubic interpolation by reconstructed areas in Table 1 shows that ScSR is particularly effective in 0_2, 1_0, 1_2, and 1_3. The areas where local gradient changes and landforms, such as small ridges are more characteristic than other areas and are thought to be the result of the advantages ScSR, which reconstructs while selecting characteristic bases from the dictionary.

Despite the effectiveness of ScSR, the proposed method is preliminary and has room for improvement, as shown below. First, although the usefulness of ScSR has been demonstrated in the Mid-Okinawa Trough area, it is necessary to verify the method in other areas to confirm its versatility. Subsequently, the size of the patch and basis was 800 m square and faults and small knolls were successfully extracted. However, it must be further verified whether it is possible to extract a more detailed topography or global structure by changing the patch size. Finally, we used grid data which were converted from the point-cloud data obtained by the MBES in this study. Although grid data can be shared easily and its size is reduced, the information included in the original point-cloud data is lost to some extent when the data are converted. The proposed method can achieve super-resolution while providing most of the information on the observation data by improving it to apply the point-cloud data.

## 6. Conclusions

In this study, we improved the ScSR proposed by [6,9]. We separated the seabed topographical image into a high-frequency component that specialises in the information on topographical undulations and a low-frequency component that captures the global information and implements sparse modelling to the high-frequency component. ScSR was effective for the super-resolution of seabed topographical maps. This method was applied to the map of the Mid-Okinawa Trough and the obtained RMSE was improved by 30% over bicubic interpolation with small training datasets. The base-extraction process and reconstruction can provide super-resolution and geoscientific interpretation simultaneously.

## Figures and Tables

**Table 1:** RMSEs for eight regions reconstructed using the dictionaries learned in the other seven other sea areas, the RMSEs for bicubic interpolation, and their ratio. The unit of RMSE is metre in this table.

Reconstruct Area	0_0	0_1	0_2	0_3	1_0	1_1	1_2	1_3	Mean
ScSR	0.803	1.183	1.156	1.853	1.193	1.259	1.414	1.723	1.323
bicubic	1.066	1.458	1.713	2.501	1.794	1.789	2.293	2.524	1.892
ScSR/bicubic	0.753	0.812	0.675	0.741	0.665	0.703	0.617	0.682	0.709

## Data Availability

Not applicable.

## Appendix A

This section describes the details on edge component extraction of the high-frequency component of training data $\tilde{Y}$ and the high-frequency component of input data Y, and its dimensionality reduction [30]. In the process of dictionary-learning, the edge components of $\tilde{Y}$ were extracted by applying first- and second-order derivative filters in both the horizontal and vertical directions.

(A1) $$\begin{array}{c} {\tilde{Y}}_{h}=Q\left(\tilde{Y}⨂f_{h1}\right),\;\;{\tilde{Y}}_{\mathrm{hh}}=Q\left(\tilde{Y}⨂f_{h2}\right),\\ \;\;{\tilde{Y}}_{v}=Q\left(\tilde{Y}⨂f_{v1}\right),\;{\tilde{Y}}_{\mathrm{vv}}=Q\left(\tilde{Y}⨂f_{v2}\right), \end{array}$$

where Q and f denote an up-sampling operator and differential filter, respectively. In this study, we adopted bicubic interpolation as the up-sampling method as mentioned in the “Method” section. The subscripts h and v denote the direction of the differential filters, and 1 and 2 are the order of the derivative. ⨂ in an operator that acts on the matrix with a differential filter. For each of ${\tilde{Y}}_{h}$, ${\tilde{Y}}_{v}$, ${\tilde{Y}}_{\mathrm{hh}},$ and ${\tilde{Y}}_{\mathrm{vv}}$, we divide them into patches of size $\sqrt{n}\times \sqrt{n}$ (where n is the patch length) to obtain a group of four matrices, each with N patches. We perform principal component analysis on the group of patches with a length of 4n and perform dimensionality reduction up to the $n_{\mathrm{low}}$-th component which has a cumulative contribution rate of 99.9% ($n_{\mathrm{low}}<4n$).

In the process of reconstruction, edge components of Y is also extracted as the same manner.

(A2) $$\begin{array}{c} Y_{h}=Q\left(Y⨂f_{h1}\right),\;\;Y_{\mathrm{hh}}=Q\left(Y⨂f_{h2}\right),\\ \;\;Y_{v}=Q\left(Y⨂f_{v1}\right),\;Y_{\mathrm{vv}}=Q\left(Y⨂f_{v2}\right). \end{array}$$

We performed principal component analysis on the obtained four-edge components and reducing the dimensionality to the $n_{L}$-th component, where the cumulative contribution was 99.9%.
